# Correlation between Vertical Transmission of Hepatitis B Virus and the Expression of HBsAg in Ovarian Follicles and Placenta

**DOI:** 10.1371/journal.pone.0054246

**Published:** 2013-01-31

**Authors:** Minmin Yu, Qian Jiang, Xiaojun Gu, Lili Ju, Ying Ji, Kaihua Wu, Hongxiu Jiang

**Affiliations:** 1 Department of Obstetrics and Gynecology, Second Affiliated Hospital of Southeast University, Nanjing, Jiangsu Province, China; 2 Department of Obstetrics and Gynecology, Yixing People’s Hospital, Yixing, Jiangsu Province, China; 3 Department of Infectious Diseases, Second Affiliated Hospital of Southeast University, Nanjing, Jiangsu Province, China; The University of Hong Kong, Hong Kong

## Abstract

**Background:**

The aim of this study was to investigate the correlation between the expression of hepatitis B surface antigen (HBsAg) in human ovary and placenta and the vertical transmission of hepatitis B virus (HBV).

**Methodology/Principal Fidnings:**

Ovarian and placental tissue specimens of pregnant women infected with HBV were collected during cesarean section and immunostained for HBsAg. The sera of the corresponding newborns were tested for HBV markers and HBV DNA. HBsAg was detected in 15 out of 33 (45%) placental tissues and was further detected in capillary endothelial cells in 4 specimens (26%), of which 3 (75%) corresponding infants were infected with HBV *in utero*. Out of the 33 ovarian tissues, 7 (21%) were positive for HBsAg, of which 2 (28%) showed HBsAg in ovarian follicles and the 2 corresponding infants (100%) had intrauterine HBV infection.

**Conclusions/Significance:**

HBsAg expression in cells of the ovarian follicle or placental capillary endothelium signal a higher risk for intrauterine HBV infection.

## Introduction

Vertical transmission of hepatitis B virus (HBV) is considered the main route of spread of HBV in areas where it is prevalent. HBV infection includes intrauterine infection, intrapartum infection, and puerperal infection. The transmission from mother to infant by the latter two modes can be blocked by treating the infant with either HBV vaccine or anti-HBV immunoglobulin immediately after birth. Despite these measures 5–10% infants fail to acquire immunity [1]–[3], which is mainly attributed to intrauterine infection. The mechanism of HBV intrauterine infection is not clear yet, but there are several hypotheses, such as transplacental transmission via serum from mother to fetus, cellular transmission by peripheral blood mononuclear cells (PBMC) [4], [5], and germ-line transmission, also called genetic transmission, in which the sperm, oocyte, or zygote *per se* is infected with HBV. Transplacental transmission is the main route of HBV transmission from mother to child [6], [7]. It was also observed that the number of HBV-infected cells decreased across the placenta from maternal end to fetal end, while the occurrence of intrauterine infection in the infant was correlated to the HBV positivity in placenta on the fetal end, i.e. the higher the rate of infected cells in placenta on the fetal end, the higher was the risk of intrauterine infection [8]–[10]. The mechanism of genetic transmission, though controversial and unclear yet, refers to a HBV-infected oocyte fertilized by a healthy sperm, that develops into an embryo in which HBV continues to replicate during embryonic development, resulting in the infant being a congenital HBV carrier, and a high risk of early onset hepatitis.

It has been reported in recent years that in hepatitis B patients, HBV can infect the sperm and HBV genomic DNA can be integrated into the genomic DNA of the sperm [11], [12]. Sperm and oocyte can be carriers of HBV and transfer viral DNA to fetus, which has been confirmed in animal models [13], [14]. However, very few studies have been done on HBV-infected human oocytes, because the human oocyte is difficult to obtain for research purposes. Recently, Ye *et al*. showed that HBsAg (hepatitis B surface antigen), HBcAg (hepatitis B core antigen), and HBV DNA were detected in ovarian tissues and may also be present in oocytes of chronic hepatitis B patients [15]–[17]. By contrast, Lou *et al*. [18] reported HBsAg in only one ovarian tissue out of 68 from pregnant women positive for serum HBsAg, but the newborn baby of this woman was negative for all tests for HBV. On the contrary, HBsAg was not detected in the ovarian tissue of the mother of a newborn with intrauterine HBV infection. Therefore, genetic transmission of HBV from infected oocyte has not been completely confirmed yet.

In the present study, we investigated the mechanism of vertical transmission of HBV, and in particular, the possibility of transmission from oocyte, in a clinical setting. We recruited pregnant women who were positive for serum HBV DNA and looked for expression of HBsAg in ovarian and placental tissues, and for HBV markers in their infants.

## Materials and Methods

### Patients

This study was conducted between January 2008 and January 2010 at the Department of Obstetrics, Second Affiliated Hospital, Southeast University, Nanjing, Jiangsu Province, China. Pregnant women who gave birth by Cesarean section, and their full-term newborns were recruited into this study. The inclusion criteria for the women were as follows: 20–40 years old, gestational period 37–42 weeks, positive for serum HBsAg and HBeAg (hepatitis B e antigen) for >6 months, and serum HBV DNA >10^6^copies/ml. Fathers of the children were not HBV carriers nor HBV hepatitis patients, were negative for serum HBsAg and HBeAg, and had negative serum HBV DNA. Hepatic function was not considered as a selection criteria. The exclusion criteria were: developmental defects in the placenta, ectopic placenta, placental abruption, abortion, premature labor, neonatal malformations, low birth weight or severe asphyxia. None of the women in the study group were treated with Hepatitis B immunoglobulin (HBIG) or antiviral drugs during pregnancy. As controls, non-HBV infected pregnant women were recruited. Women in either the test or control groups were admitted to the study if they had ovarian cysts or solid masses considered as benign, or malignant ovarian tumors detected by ultrasonography or confirmed during operation, requiring excision of the lesion or ovary. From all recruited women, placental and ovarian tissue samples of dimensions 1 cm×1 cm×2 cm, with intact anatomical layers, were collected during surgery. The study was approved and authorized by the Institutional Review Board of the Second Affiliated Hospital of Southeast University, Nanjing 210003, China. Written informed consent was obtained from every participant (Approval ID. 2008-LL-008).

### HBsAg Detection by Histochemical Methods

The ovarian and placental tissue samples were processed by routine histological procedures and paraffin sections were prepared by the standard series of operations of fixation, dehydration and paraffin embedding. The slides were then stained with hematoxylin-eosin (HE) and observed using light microscopy to evaluate the histology.

HBsAg was detected in ovarian and placental tissues using an immunohistochemical staining kit containing a mouse anti-HBsAg monoclonal antibody and a secondary antibody linked to peroxidase with streptavidin-biotin amplification, following the procedure recommended by the manufacturer (SP method, U.S. Biologicals, Massachusetts, USA). In detail, the paraffin-embedded tissue slices were dewaxed and rehydrated by passing through a gradient of alcohol by the conventional method. The slices were rinsed 3 times for 3 min each with phosphate-buffered saline (PBS) diluted 1∶50 in water, and incubated with 50 µL peroxidase blocking fluid at room temperature for 10 min, followed by PBS rinses as described above. The slides were incubated with 50 µL non-immunized mouse serum at room temperature for 10 min, followed by one rinse in PBS and then incubated with mouse anti-HBsAg monoclonal antibody at room temperature for 60 min, followed by 3 PBS rinses of 3 min each. Next, the slides were incubated with 50 µL streptavidin peroxidase at room temperature for 10 min, followed by PBS rinses as described above. Diaminobenzidine (DAB; U.S. Biologicals, Massachusetts, USA) was used to visualize the immune reaction and slides were counterstained with hematoxylin. Lastly, the slides were treated with xylene and ethanol for improving transparency, and fixed with neutral gum. The slides were observed using light microscopy. HBsAg staining was considered positive when the cell membrane or cytoplasm was brownish-yellow in color. An internal control was created by replacing mouse anti-HBsAg antibody with PBS in the protocol described above. Slides from HBV patient samples were also compared with those from non-HBV volunteers following anti-HBsAg staining. Hepatic tissues from HBV hepatitis patients were subjected to anti-HBsAg staining as positive control.

### Immunization of Newborns and Follow-up

4 ml of blood from the femoral vein was taken immediately after birth for detection of serum HBV markers (HBVM,including HBsAg,Anti-HBs,HBeAg, Anti-HBe,Anti-HBc) and HBV DNA. Newborns who were HBV positive were immunized with 20 µg of recombinant hepatitis B vaccine prepared by yeast gene engineering technology (HBVac, North China Pharmaceutical Corp. Inc., Shijiazhuang, Hebei Province, China, 10 µg), and 200 IU HBIG (Shu Yang Pharmaceutical, Chengdu, Sichuan province, China). Newborns were immunized again at the age of 1 month and 6 months with 20 µg HBVac, while HBIG treatment was repeated once at the same dose 15 days later. HBVM and HBV DNA were measured once again at age of 1 month and 7 months.

### Serum HBV DNA and HBVM Measurement

Serum HBV DNA was detected by quantitative real-time PCR using a kit (Shanghai Biological Engineering Co., Ltd., Shanghai, China). The sensitivities of the kit was 5.0×10^2^–1.0×10^9^ copies/mL. HBVM was detected using a chemiluminescence method (American Abbott, USA). Procedures were carried out strictly according to the instructions from the manufacturers.

### Diagnostic Criteria of HBV Intrauterine Infection

Newborns who were positive for neonatal serum HBsAg and/or HBV DNA for 7 months were diagnosed as having intrauterine HBV infection.

### Statistical Analysis

SPSS13.0 statistical software was used in this study. χ^2^ test or Fisher’s exact test were applied for categorical data analysis. Data were considered significant if P<0.05.

## Results

Only 33 of 3650 (0.90%) HBV-positive pregnant women met the study selection criteria and were included in the study. A further 5 of 574 (0.87%) HBV- negative pregnant women met the study criteria and were recruited as controls. There was no statistically significant differences between the two groups at a level of P>0.05. In total, 33 placental and ovarian tissue samples were collected at the time of Caesarean section, and shortly after delivery blood samples were collected from the corresponding infants. The tissue samples were analyzed for the presence of HBsAg, while the blood samples were analyzed for presence of HBV.

### Incidence of HBV Intrauterine Infection

All the mothers (n = 33) were HBV-positive for at least 6 months with a mean logarithmic HBV DNA level of 7.74±0.56 copies/mL. Out of the 33 newborns, 6 had HBV infection at birth, and 4 infants continued to be positive for HBV DNA at 7 months of age with a mean logarithmic value of 6.23±1.88 copies/mL HBV DNA. Thus, the incidence of HBV intrauterine infection in the infants was 12.12% (4/33). The corresponding maternal ovarian and placental tissues for these infants were further analyzed for expression of HBsAg.

### Correlation between HBsAg Expression Pattern in Ovary and Intrauterine Infection

**Figure 1:**
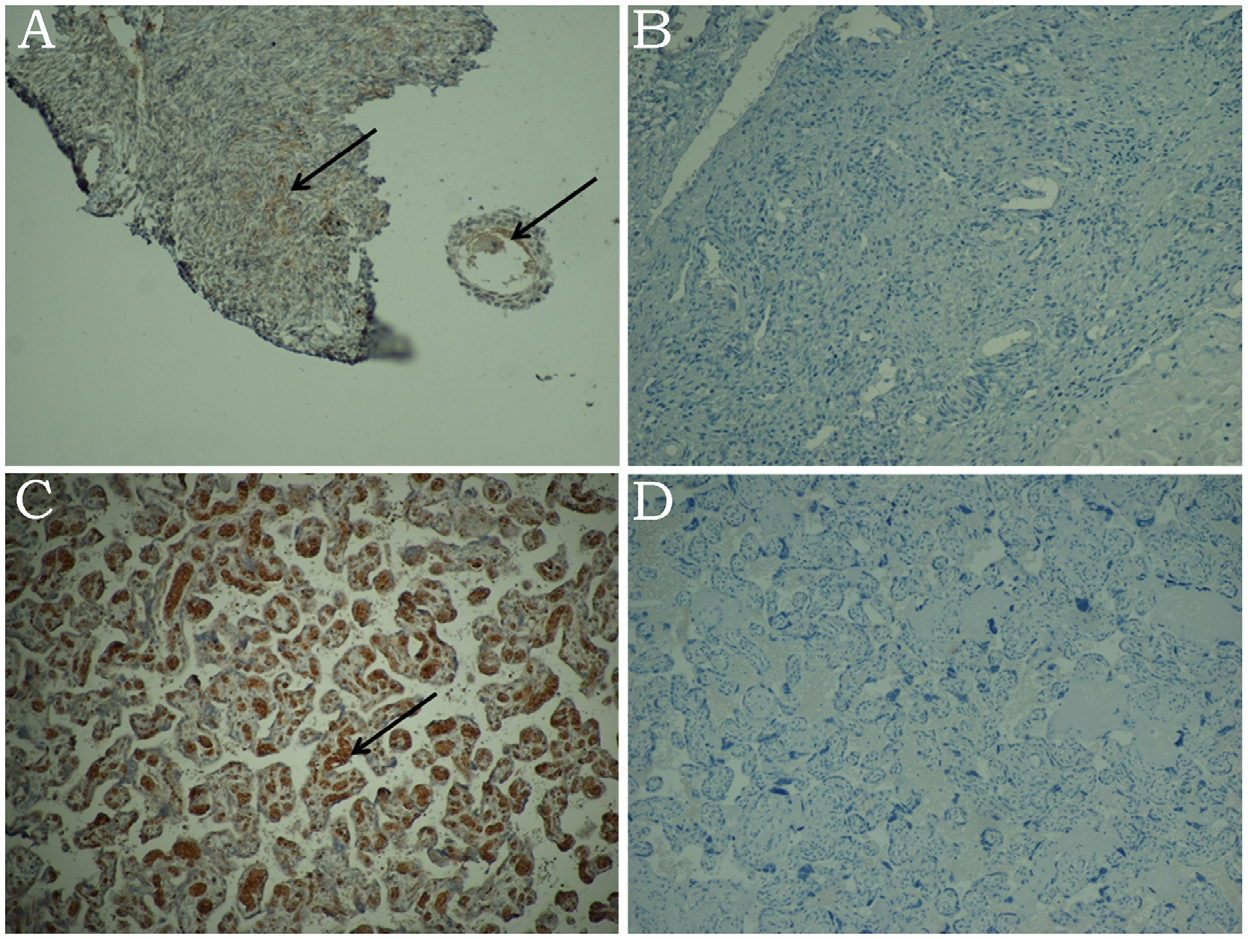
Immunostaining for HBsAg in ovary and placenta in a HBV-positive woman and a non-HBV control specimens. A: Positive HBsAg immunostaining in oocyte and ovarian intercellular matrix in a HBV-positive woman. Magnification: 10×10. B: Negative HBsAg immunostaining in ovarian intercellular matrix in a non-HBV control woman. Magnification: 10×10. C: Positive HBsAg immunostaining in placenta in a HBV-positive woman. Magnification: 10×20. D: Negative HBsAg immunostaining in placenta in a non-HBV control woman. Magnification: 10×20.

All the 33 women recruited in the study had benign ovarian tumors or lesions. HBsAg expression in the ovaries was detected in 7 patients (7/33, 21.21%). In 2 out of these 7 women (2/7, 28.57%), the expression of HBsAg was widespread in the ovaries: in follicles, ovarian intercellular matrix, and corpus luteum. HBsAg was mainly detected in the cytoplasm of oocytes and stromal cells (Figure 1A). Out of the 7 women positive for HBsAg in the ovaries and having ovarian cysts considered as benign, 3 had children with confirmed intrauterine infection (3/7, 42.85%). Out of the 33 participants, 26 showed no immunostaining for HBsAg in the ovaries and the infant of one of these 26 women (3.85%) had confirmed intrauterine HBV infection. The incidence of intrauterine infection was analyzed with respect to the localization of HBsAg in the ovaries (Table 1). Infants of both the women who had widespread HBsAg immunostaining in the ovaries, i.e., in the follicles as well as intercellular spaces, had confirmed intrauterine HBV infection (Table 1). A significant difference was observed in the occurrence of intrauterine infection between those who showed HBsAg immunostaining in follicle and intercellular substance, and those who did not (χ^2^ = 17.949, P = 0.008).

**Table 1:** Correlation between HBsAg expression pattern in maternal ovary and occurrence of intrauterine infection in the infant.

	Positive in both follicle and intercellular substance (Group A)	Follicle negative Intercellular substance positive (Group B)	Negative in both Follicle and intercellular substance (Group C)
No. of cases (n)	2	5	26
Intrauterine infection	100.00% (2/2)	20.00% (1/5)	3.85% (1/26)

Out of the 33 samples of ovarian tissues examined, 7 were positive for immunostaining for HBsAg.

Comparison of intrauterine infection rate between Group A and Group B : χ^2^ = 3.733, P = 0.143.

Comparison of intrauterine infection rate between Group A and Group C: χ^2^ = 17.949, P = 0.008.

Comparison of intrauterine infection rate between Group B and Group C: χ^2^ = 1.813, P = 0.301.

HBsAg staining was negative in the five non-HBV controls (Figure 1B), and none of their infants had intrauterine HBV infection. HBsAg staining was positive in hepatic specimens from 5 HBV hepatitis patients.

### Correlation between HBsAg Expression Pattern in Placenta and Intrauterine Infection

**Figure 2:**
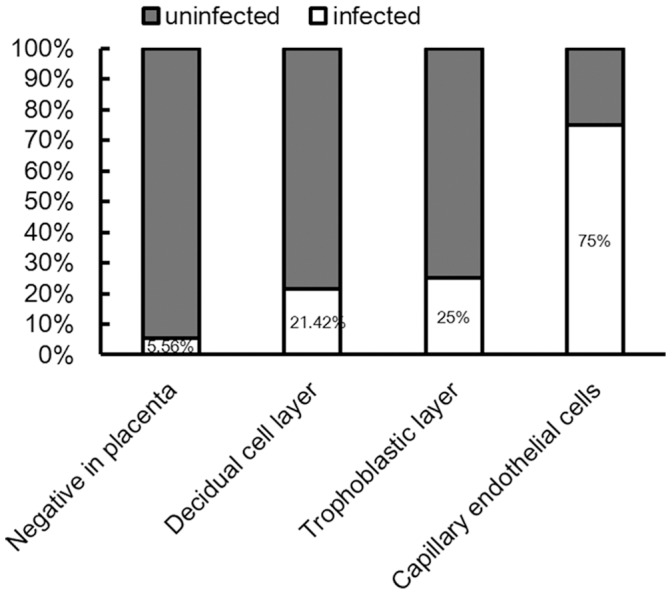
Increasing risk of intrauterine infection with progression through the placental layers.

Out of the 33 women included in the study, 15 women (45.45%, 15/33) had positive immunostaining for HBsAg in the placenta (Figure 1C). Out of these 15 women, infants of 3 women had confirmed intrauterine HBV infection (3/15, 20%). Eighteen women were negative for HBsAg immunostaining in the placenta and only one infant in this group had confirmed intrauterine HBV infection (1/18, 5.56%). The rate of intrauterine HBV infection in the infants was not significantly different between the groups of patients who were positive for HBsAg in placenta and those who were negative for HBsAg in placenta (χ^2^ = 1.603, P = 0.308). The incidence of intrauterine HBV infection in the infants was analyzed in terms of localization of HBsAg immunostaining across the placenta, from the maternal end to the fetal end: decidual cell layer, trophoblastic layer and capillary endothelial cells. The number of women with positive HBsAg immunostaining in these locations decreased in the order decidual cell layer, trophoblastic layer and capillary endothelial cells, whereas, the corresponding risk of intrauterine infection increased in the same order (Table 2, Figure 2). HBsAg were detected in capillary endothelial cells in 4 specimens (4/15, 26.67%), of which 3 (3/4,75%) of the corresponding infants had intrauterine HBV infections. Thus, intrauterine HBV infection rate in infants was much higher if the mother was positive for HBsAg immunostaining in capillary endothelial cells than if the mother was negative for HBsAg in capillary endothelial cells (χ^2^ = 10.610, P = 0.0098), or in any other layer of the placenta (χ^2^ = 10.313, P = 0.0088).

**Table 2:** Correlation between HBsAg expression pattern in maternal placenta and the occurrence of intrauterine infection in infants.

	Decidual cell layer		Trophoblastic layer		Capillary endothelial cells		Negative in placenta
	Positive	Negative	Positive	Negative	Positive	Negative	
No. of cases (n)	14	1	8	7	4	11	18
Intrauterine infection (%)	3/14(21.42)	0/1(0)	2/8(25)	1/7(14.28)	3/4(75)	0/11(0)	1/18(5.56)

Out of the 33 samples of placental tissues examined, 15 were positive for immunostaining for HBsAg.

Comparison of intrauterine infection rate between HBsAg positive and negative in decidual cell layers: χ^2^ = 0.268, P = 0.8000; comparison between HBsAg positive in decidual cell layers and negative in placenta: χ^2^ = 1.814, P = 0.1822.

Comparison of intrauterine infection rate between HBsAg positive and negative in trophoblastic layer: χ^2^ = 0.268, P = 0.4308; and between HBsAg positive in trophoblastic layer and negative in placenta: χ^2^ = 2.052, P = 0.1938.

Comparison of intrauterine infection rate between HBsAg positive and negative in capillary endothelial cells: χ^2^ = 10.313, P = 0.0088; and between HBsAg positive in capillary endothelial cells and negative in placenta: χ^2^ = 10.610, P = 0.0098.

Comparison of intrauterine infection rate between HBsAg positive in decidual cell layer and trophoblastic layer: χ^2^ = 0.037, P = 0.3870; and between HBsAg positive in decidual cell layer and capillary endothelial cells: χ^2^ = 4.018, P = 0.07843.

Comparison of intrauterine infection rate between HBsAg positive in trophoblastic layer and capillary endothelial cells: χ^2^ = 2.743, P = 0.1414.

HBsAg staining in the placenta was negative in the 5 non-HBV controls (Figure 1D) and their infants had no intrauterine HBV infection. Compared with patients who were positive for serum HBsAg, the intrauterine HBV infection rate in these infants was not significantly different from the non-HBV controls (χ^2^ = 0.677, P = 1.0).

In this study, 4 out of the 33 women had infants with confirmed intrauterine HBV infection. Of these 4, there was HBsAg expression in both, ovarian follicle and placenta in one woman, only in the ovarian follicle in one woman, and only in the placenta in two women (Table 3).

**Table 3:** HBsAg expression in ovarian follicle and placenta of 4 patients whose newborns had intrauterine HBV infection.

	Ovarian Follicle HBsAg	Placenta HBsAg
Case 1	+	+
Case 2	+	−
Case 3	−	+
Case 4	−	+

## Discussion

HBV was long regarded as a specifically hepatotropic virus, but is now recognized as a pantropic virus as it has been found in extrahepatic tissues and organs in many studies [19]–[23]. However, whether HBV can infect ovaries, and persist and replicate within ovarian tissue has been rarely studied in China or internationally. In this study, we examined ovarian and placental tissues of HBV-infected women who were referred to the obstetrics department and monitored the occurrence of intrauterine HBV infection in the newborns. Serum HBV DNA levels in pregnant women had already been an independent risk factor for viral transmission to infants, but might also be correlated with widespread HBsAg expression in reproductive and other tissues. To increase the percentage of positive ovarian and placental tissues, all the women selected had serum HBV DNA >10^6^copies/mL and the measured mean logarithm HBV DNA level of the 33 women in the study was 7.74±0.56 copies/mL.

HBsAg, the surface antigen, is encoded by the S gene and is one of the highly immunogenic epitopes of HBV. HBsAg is therefore the target of choice for several methods for detection of the virus, including those based on immunohistochemistry. Obtaining a single oocyte for *in situ* detection of HBV infection is no doubt the most ideal and straight forward strategy to monitor HBV transmission through germ cells; however, obtaining an oocyte is very difficult and this has not been attempted. On the other hand, ovarian tissue is much more accessible and can be examined by immunohistochemical methods for HBsAg.

In recent years, Ye *et al.* [15]–[17] have reported that HBsAg, HBcAg, and HBV DNA persisted in ovaries of female hepatitis B patients. Moreover, these markers were found in the oocyte as well, which was the most direct evidence of HBV infection of germ cells. However, so far there is no report of HBV infection in ovaries during pregnancy. In the current study, 33 pregnant women positive for serum HBsAg were recruited, and their ovarian and placental tissues were obtained at the time of delivery. Out of 33 women, 7 women (21.21%) were confirmed to have HBV infected ovaries, of which 2 women showed positive HBsAg staining in oocytes, intercellular matrix, and corpus luteum. Most positive staining was found in the cytoplasm, which is a reservoir of de novo synthesized HBV. Presumably, the route of infection of the ovaries is from the liver via the bloodstream. In the current study, ovarian tissue specimens were obtained from women who had HBV infection and also carried benign ovarian tumors or lesions. It has been confirmed that HBV infection was correlated closely with hepatocellular carcinoma and damage of extrahepatic tissues and organs [21], [24], but whether HBV infection of the ovary leads to histological changes or results in benign tumors was not addressed in this study and requires further exploration.

HBV has been shown to infect all layers of the placenta, including decidual, trophoblastic, and capillary endothelial cells [6], [7]. In the current study, 15 out of 33 women (45.45%) had placenta infected with HBV, which was significantly higher than the incidence of HBV infection in the ovaries. The findings that HBsAg staining across the placenta decreased gradually from the maternal end to fetal end, and that HBsAg was mainly localized to the cell cytoplasm, are consistent with previous reports [8]–[10].

Although there has been accumulating evidence of HBV transmission from mother-to-child in recent years, the mechanism of vertical transmission via oocyte is still poorly understood. Animal models have demonstrated that the sperm and oocyte can be carriers of HBV, and can transfer virus into embryos [13], [14]. By contrast, Lou *et al.* [18] suggested that the intrauterine infection rate in infants of women positive for serum HBsAg is not correlated with the positive staining of HBsAg in ovaries. In the present study, this rate was 42.85%, as 3 infants were HBV-positive out of the 7 infants whose mothers had HBsAg in the ovaries. Both the mothers who had HBsAg staining in oocytes gave birth to intrauterine infected infants, so that the infection rate was 100%, which is significantly higher than women who had HBsAg positive staining only in intercellular matrix or those who were negative for HBsAg in ovaries (P<0.01). This suggests that the risk of vertical transmission of HBV is largely determined by the location of HBsAg expression; infection in oocytes might present a high risk of transmission of HBV to fetus. More importantly, one of the 2 oocyte-HBV-infected patients had no HBsAg staining in placenta, so that the possibility of intrauterine infection from placenta might be very small and this might be evidence of HBV vertical transmission from oocytes in humans. Another oocyte infected patient on the contrary had positive staining of HBsAg in placenta. Therefore the intrauterine infection seen in the corresponding infant might be a synergetic effect of the two possible routes of transmission, from the placenta and oocyte. The intrauterine infection rate was 20% in patients positive for HBsAg in placenta, higher than that in those negative for HBsAg in placenta, though the difference was statistically was not significant (P>0.05). However the lack of placental samples would make the outcomes contrary to other studies, so it requires further studies with a larger samples sets. Also, whether the infants had characteristics that precluded HBV vaccination efficacy and whether the placental HBV was the same at birth as it was during the whole pregnancy is not yet known. These factors might have an effect on the results. Also, occurrence of HBsAg immunostaining across the placenta decreased from the maternal end to fetal end, however, positive HBsAg staining in the fetal end of the placenta indicated higher risk of intrauterine infection. For instance, the intrauterine infection rate in the patients having HBsAg positive staining in vascular endothelial cells was significantly higher than that in those negative for this type of staining (χ^2^ = 10.313, P = 0.0088); and was also higher than that in those negative for staining in placenta (χ^2^ = 10.610, P = 0.0098). Therefore, HBsAg positive staining in placental vascular endothelial cell is an important index of intrauterine infection. In this study, 3 out of the 4 intrauterine infections had HBsAg positive staining in placenta, indicating that intrauterine HBV infection is mainly transplacental, while transmission via oocyte is a secondary route with lower probability.

Our findings have implications for blocking vertical transmission of HBV in clinical practice. A similar study on a larger population will be required to clarify the mechanisms and risk factors of intra-uterine HBV infections.
